# Palms, peccaries and perturbations: widespread effects of small-scale disturbance in tropical forests

**DOI:** 10.1186/1472-6785-12-3

**Published:** 2012-03-19

**Authors:** Simon A Queenborough, Margaret R Metz, Thorsten Wiegand, Renato Valencia

**Affiliations:** 1 Department of Evolution, Ecology and Organismal Biology, The Ohio State University, Columbus, OH 43210, USA; 2 Department of Plant Pathology, University of California, Davis, CA 95616, USA; 3 Department of Ecological Modelling, UFZ Helmholtz Centre for Environmental Research-UFZ, PF 500136, D-04301 Leipzig, Germany; 4 Laboratory of Plant Ecology, School of Biological Sciences, Pontifical Catholic University of Ecuador, Quito, Ecuador

## Abstract

**Background:**

Disturbance is an important process structuring ecosystems worldwide and has long been thought to be a significant driver of diversity and dynamics. In forests, most studies of disturbance have focused on large-scale disturbance such as hurricanes or tree-falls. However, smaller sub-canopy disturbances could also have significant impacts on community structure. One such sub-canopy disturbance in tropical forests is abscising leaves of large arborescent palm (Arececeae) trees. These leaves can weigh up to 15 kg and cause physical damage and mortality to juvenile plants. Previous studies examining this question suffered from the use of static data at small spatial scales. Here we use data from a large permanent forest plot combined with dynamic data on the survival and growth of > 66,000 individuals over a seven-year period to address whether falling palm fronds do impact neighboring seedling and sapling communities, or whether there is an interaction between the palms and peccaries rooting for fallen palm fruit in the same area as falling leaves. We tested the wider generalisation of these hypotheses by comparing seedling and sapling survival under fruiting and non-fruiting trees in another family, the Myristicaceae.

**Results:**

We found a spatially-restricted but significant effect of large arborescent fruiting palms on the spatial structure, population dynamics and species diversity of neighbouring sapling and seedling communities. However, these effects were not found around slightly smaller non-fruiting palm trees, suggesting it is seed predators such as peccaries rather than falling leaves that impact on the communities around palm trees. Conversely, this hypothesis was not supported in data from other edible species, such as those in the family Myristicaceae.

**Conclusions:**

Given the abundance of arborescent palm trees in Amazonian forests, it is reasonable to conclude that their presence does have a significant, if spatially-restricted, impact on juvenile plants, most likely on the survival and growth of seedlings and saplings damaged by foraging peccaries. Given the abundance of fruit produced by each palm, the widespread effects of these small-scale disturbances appear, over long time-scales, to cause directional changes in community structure at larger scales.

## Background

Disturbances are an important process structuring forests worldwide and have long been considered as significant drivers of dynamics and diversity [1-3]. In tropical forests, disturbances such as hurricanes and tree-falls from lightning or wind events create a mosaic of forest patches of different microhabitats at varying stages of succession [4-6] superimposed upon background topographical and soil variation. If niche partitioning permits coexistence along the axis of tolerance to disturbance, different species should be selected by such disturbances, and we would expect to find predictable suites of species associated with different disturbance regimes [7]. This is indeed the case. Because of a trade-off between growth and survival, fast-growing pioneer species occur predictably in tree-fall gaps. Conversely, slow-growing shade-tolerant species survive well in closed canopy forest [8-10]. Furthermore, resprouting of trees is high in areas affected by hurricanes [11]. These large-scale disturbances are obvious in the forest and are important mechanisms of species coexistence and community structure [4,12]. However, the contribution of gaps and their associated pioneer flora to species coexistence is likely to be limited, because full tree-fall gaps are relatively infrequent in lowland rain forest [13] and most pioneer species occur at low abundance [12]. Are there other kinds of disturbance in tropical forests affecting far wider areas of forest that influence species composition and community dynamics?

Several authors have suggested that smaller disturbances such as branch-falls have a greater and more widespread effect on forest dynamics and composition than might be apparent at first glance [13-15]. In the absence of large canopy-opening disturbances, mortality in sub-canopy layers can increase light availability, free up resources and lead to faster sapling growth [16,17]. Furthermore, these sub-canopy gaps occupy large proportions of forest [13]. Sub-canopy openings occur from a variety of processes, including the death of sub-canopy trees, large branch falls, or in-filling of canopy gaps by trees in middle forest strata [14]. In addition to freeing resources, these events may cause disturbances to younger life stages, such as branch-falls affecting the survival and growth of seedlings and saplings [18-21]. Falling branches are heavy and can kill or physically damage small plants [22]. If these branch-falls were prevalent enough of a selective force, species intolerant of this damage could be selected against in areas of high branch-fall. Peters et al. [15] extend this argument to apply to arborescent palm leaves, which are heavy enough to physically damage anything underneath (> 15 kg dry mass and several metres in length [23]). Given the ubiquity of large palms in western Amazonian forests (densities often exceeding 60 palms > 10 cm dbh ha^-1 ^[24-29]), these authors suggested that falling leaves could select for species adapted to withstand this disturbance impact, such as those with storage organs or cotyledons that could easily resprout following damage. Indeed, Peters et al. [15] found differences in sapling (0.5-2.5 m in height) communities in areas close to large arborescent palms compared to those with no influence of palm leaf fall. Although theirs was only a small study over 2.25 ha, it would seem that falling palm leaves could have a large effect on the species composition of forest communities.

However, falling branches and leaf fronds are not the only widespread small-scale disturbances in forests. Large frugivores such as tapirs and peccaries are known to cause heavy localised disturbance, dramatically increase mortality of seedlings and saplings [30-33], and consume and disperse seeds [34,35]. Peccaries also consume palm fruit [33] and in a repeat study of Peters et al. [15], Beck et al. [36] showed that sapling communities were impacted by the presence of palms in forest containing peccaries, but not in forest where they had been hunted out. Beck et al. [36] attributed the relationship between palms and saplings to peccaries rooting around palms and not to falling palm fronds. However, both studies [15,36] suffered from using only static data, examining distributions of palms relative to other species without the ability to test how these distributions develop through differential growth or mortality.

Whatever the mechanism (falling fronds or rooting peccaries), the disturbance around palms should cause differences in seedling and sapling density, growth, mortality, and/or re-sprouting close to palms compared to areas further away. If falling fronds are the cause, there should be similar effects in the neighbourhoods of large, fruiting palms as well as large, but non-fruiting palms. Alternatively, if the disturbance is caused by peccaries, we would not expect an effect by non-fruiting palms. In addition, although palms are a common food source, many other species of large-fruited tree are also consumed by peccaries [33]. Therefore, we would also expect an effect on the seedling and sapling communities around other large fruit-bearing tree species similarly eaten by peccaries. In this latter case, the effect of these small-scale disturbances may be even more widespread than Peters et al. [15] envisaged.

We should also consider that palms may have positive effects on some species. Evidence gathered by Connell et al. [13] showed that smaller sub-canopy disturbances led to faster growth of understorey saplings, presumably because of increased light penetration or freed-up resources from the death of a sub-canopy individual. These sub-canopy gaps (without complete opening of the forest canopy) were very common in the forest (covering up to about 48% of forest). Falling palm fronds likely create such sub-canopy gaps and seedlings that survive palm or peccary damage may benefit from having (i) fewer competitors (seen in the lower local densities of seedlings and saplings), (ii) more light (because palms have a less dense canopy than other trees and because the falling fronds knock other branches down) and, (iii) more soil resources (for the same reasons). Ultimately, this increased availability of resources adjacent to palms could lead to higher growth or survival of saplings of some species.

In this study we use spatially-explicit data from long-term monitoring of seedlings, saplings in the neighbourhoods of large arborescent Arecaceae (mostly *Iriartea deltoidea*) and reproductive Myristicaceae trees in a large forest dynamics plot in western Amazonia to examine the potential structuring effect of falling leaves and branches on tropical forest communities. We aim to test (i) whether large palms and other fruiting trees appear to impact seedling and sapling dynamics and distributions and (ii) whether any difference is due to the trees themselves or an interaction with large frugivores. We extend previous studies of these phenomena in two key areas. First, we use dynamic data to understand the processes that lead to the patterns observed in static data in previous studies. Second, we consider that small-scale disturbances and/or interactions with frugivores must also apply to other fruiting trees. In this case, we examined seedlings and saplings in the neighbourhood of fruiting adults in a common dioecious family at our site, the Myristicaceae, fruits of which are also often consumed by peccaries [33]. The Myristicaceae have been studied here for 10 years, and detailed information is available on the sex of each reproductive tree. Comparing the sapling communities around male (non-fruiting) and female (fruiting) trees allows a rigorous comparison of fruit-eating by peccaries, and a counterpoint to understanding the impacts of disturbances associated with arborescent palms.

## Results

A total of 1,673 mature arborescent palms (≥ 15 cm dbh) were recorded in the 25 ha Yasuní FDP in the initial census (a mean of 67 ha^-1^, Table 1). Exactly 309 large arborescent palms in the size class 10-15 cm dbh were recorded. These palms were presumed to be immature (i.e. non-fruiting) but still with large leaves capable of causing physical damage to seedlings and saplings beneath. A total of 54,406 non-palm saplings (1-2 cm dbh) were recorded on the plot and 46,853 of these survived to the next census. A total of 12,309 new seedling recruits were recorded in the 339 seedling plots from 2003 to 2007. Fewer than half of these (4,480) survived to the next year.

**Table 1:** Abundances of the four arborescent palm species on the Yasuní FDP in the first census

Species	DBH class (cm)		
	1-10	10-15	≥ 15
*Iriartea deltoidea *Ruiz & Pav.	493	292	1520
*Astrocaryum chambira *Burret	0	3	94
*Oenocarpus bataua *Mart.	0	3	98
*Socratea exorrhiza *(Mart.) H. Wendl.	15	11	3

### Spatial patterns of distribution and density

**Figure 1:**
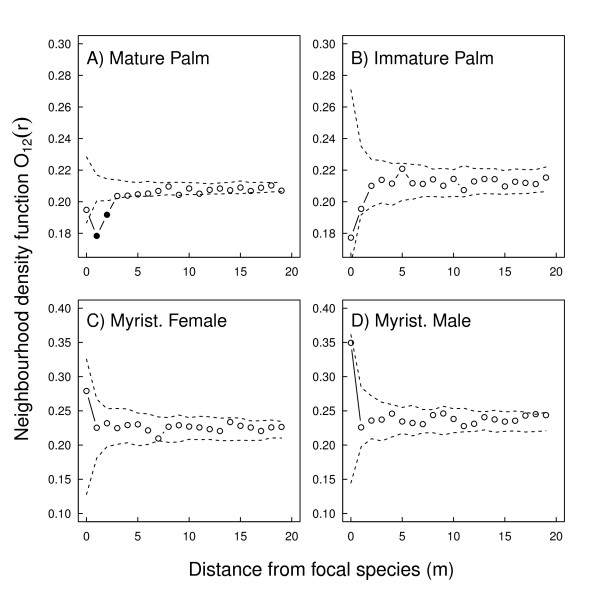
**Spatial pattern analysis of non-palm saplings compared to palm trees and Myristicaceae trees**. Results of bivariate point pattern analyses between mature (**A**, > 15 cm dbh) or immature (**B**, non-fruiting, 10-15 cm dbh) large arborescent palms, and non-palm saplings (1-2 cm dbh), and female (**C**, fruiting) or male (**D**, non-fruiting) Myristicaceae and non-palm saplings in the 25 ha Yasuní FDP. In each case we randomised the focal individuals (palms or Myristicaceae) with a heterogeneous Poisson distribution (20 m). Each pane shows the O-ring statistic (circles, *O_12_(r)*), giving the local neighborhood density of the pattern at scale r. Simulation envelopes (dashed lines) are the 5th highest and lowest O(r) of 199 randomizations of the pattern over the study region. Points lying outside of the confidence envelope (filled circles) indicate significant attraction (above) or repulsion (below) of saplings relative to palms.

In initial neighbourhood analyses using a bivariate form of the O-ring statistic with a homogeneous Poisson null model we found a significant large-scale effect, indicating that large palms are located in areas of somewhat lower sapling density (data not shown). When we corrected for this effect using the heterogeneous Poisson null model, randomly displacing palms within a 30 m neighbourhood, we found, as expected, notable small-scale repulsion of saplings from mature palms at distances < 3 m (Figure 1A). Considering immature palms, the large scale effect with respect to saplings existed as a tendency but was not significant and there were no significant small-scale effects (Figure 1B). Saplings were neither repulsed from or attracted to immature palm trees. When we extended these analyses to other fruiting trees, contrary to our predictions, there was no significant repulsion or attraction of saplings from neither male nor female Myristicaceae trees and the observed O-ring values remained within the simulation envelopes (Figure 1C and 1D).

### Spatial patterns of community structure

**Figure 2:**
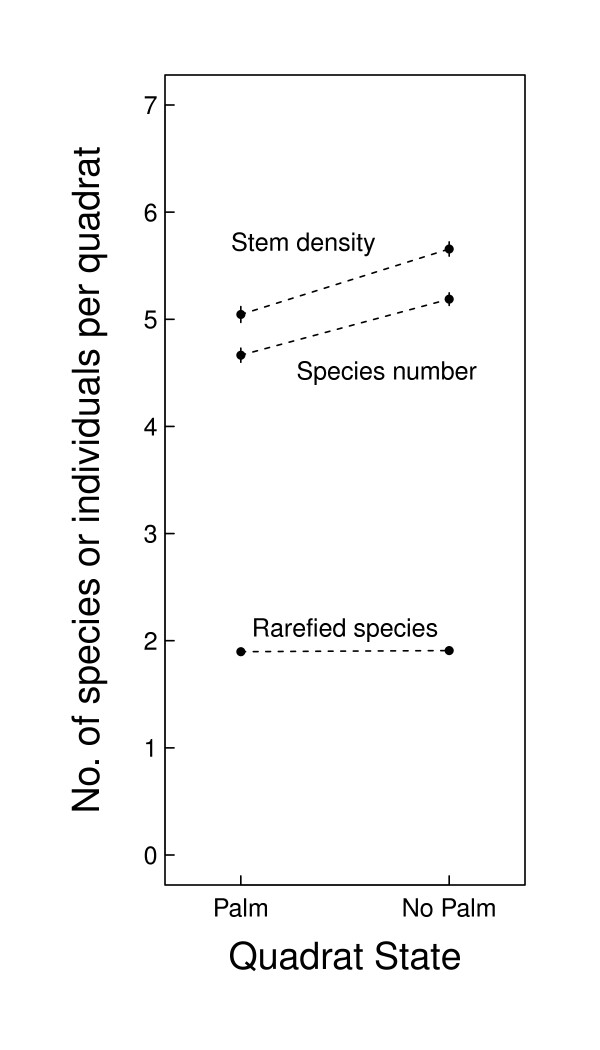
**Comparison of sapling communities in areas influenced and not influenced by large arborescent palm trees**. We show the sapling density (stem density 25 m^-2^), observed number of species (species number 25 m^-2^), and rarefied species richness (species per two individuals) for 5 × 5m quadrats that contain or are adjacent to a large palm (N = 1,471), and quadrats that are > 1 quadrat from a palm quadrat (N = 8,301). We show the mean ± SE.

A total of 1,471 5 × 5 m quadrats contained or were adjacent to a quadrat containing a mature palm ('palm quadrats') and the remaining 8,301 quadrats were defined as uninfluenced by mature palms ('non-palm quadrats', Figure 2). Both stem density and species richness were lower in palm quadrats, by about 0.5 of a stem/species. When we accounted for stem density, rarefied species richness was equivalent between palm and non-palm quadrats, indicating that the lower observed species riches in palm quadrats was primarily caused by a fewer individuals.

Testing for differences in community composition at the quadrat scale, we found a significant difference between palm and non-palm quadrats (adonis, df = 1, SS = 3.40, MSS = 3.40, F = 7.46, *R*^2 ^= 0.001, *P *< 0.001), indicating that the assemblages of species in the neighborhoods of palms differed significantly from non-palm neighborhoods, despite similar levels of diversity. However, the amount of variation explained was very low, with an *R*^2 ^of 0.001. A comparison of the abundance of species within palm and non-palm quadrats indicated that only a minority of the over 1,000 species in the plot showed a significant response to palms (Table 2). Ten species were more abundant than expected in palm quadrats, and 25 species were more abundant than expected in non-palm quadrats.

**Table 2:** Abundances of tree species with significantly high or low relative abundances in areas influenced by large arborescent palms on the Yasuní FDP in the first census

	Abundance in quadrats:		
Species	Non-palm	Palm	Proportion in non-palm
***Acalypha cuneata***	252	64	0.80
*Acidoton nicaraguensis*	862	117	0.88
***Aniba guianensis***	40	14	0.74
***Bertiera guianensis***	5	4	0.56
*Calyptranthes bipennis*	66	4	0.94
*Calyptranthes sedosa*	66	4	0.94
*Discophora guianensis*	64	3	0.96
*Endlicheria *'dori'	74	4	0.95
*Eugenia pusilliflora*	216	18	0.92
*Garcinia brasiliensis*	40	0	1.00
*Geonoma aspidiifolia*	721	71	0.91
*Guarea fistulosa*	349	37	0.90
*Guatteria scalarinervia*	95	7	0.93
*Hyospathe elegans*	120	11	0.92
*Inga auristellae*	635	86	0.88
*Leonia glycycarpa*	173	15	0.92
*Licania nervifina*	65	3	0.96
*Lacistema nena*	41	0	1.00
***Matisia oblongifolia***	1537	322	0.83
***Miconia multispicata***	36	16	0.69
***Miconia tipica***	204	54	0.79
*Mollinedia killipii*	89	4	0.96
*Neea *'bajio'	197	14	0.93
***Otoba glycycarpa***	44	16	0.73
***Pentagonia spathicalyx***	28	12	0.70
*Picramnia *'mini'	30	0	1.00
*Piper *'obchic'	1702	183	0.90
*Pourouma bicolor*	550	67	0.89
*Pouteria trilocularis*	45	1	0.98
*Sorocea muriculata*	262	26	0.91
***Tococa guianensis***	1	6	0.14
*Unonopsis veneficiorum*	330	33	0.91
Hippocrateaceae 'atenumembra'	179	18	0.91
*Eugenia *'smedcomun'	195	20	0.91
**Solanaceae 'plata'**	3	4	0.43

Species in bold are those with more individuals than expected in quadrats close to palm trees. All other species have more individuals than expected in quadrats far from palm trees.

**Figure 3:**
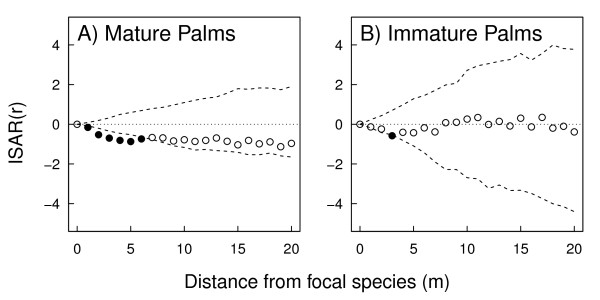
**Spatial analysis of the species diversity around large palm trees**. Individual species area relationship (ISAR) for arborescent palms in the Yasuní FDP from the first census. We used a heterogeneous Poisson null model which accounts for some degree of aggregation in the focal species. We show ISARs for (**A**) mature large palms and (**B**) immature large palms. Each pane shows the ISAR statistic for each distance (circles). Confidence envelopes are the 5th highest and lowest ISAR of 199 randomizations of the patterns over the study region. Points lying outside of the confidence envelope (filled circles) indicate significant 'diversity accumulator' (above) or 'diversity repeller' (below) with respect to the number of neighbouring sapling species.

These results were confirmed in *ISAR *neighbourhood analyses, were we found a significant repelling effect of large arborescent mature palms on surrounding species at distances < 5 m (Figure 3A). This result implies that mature palms have a limited direct negative effect on the diversity of surrounding sapling species. In contrast, immature arborescent palms had no such effect on surrounding species diversity, and the observed ISAR values for the null model generally remained within the simulation envelopes, indicating a significant but very small repelling effect at only one distance category (3 m, Figure 3B).

### Dynamic demographic effects of large trees on seedlings

**Figure 4:**
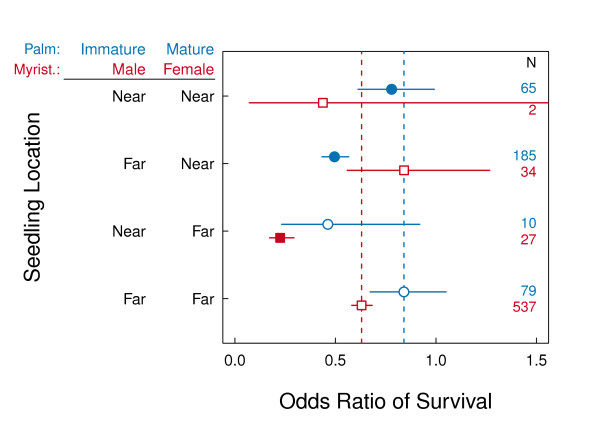
**Seedling survival around large palm trees**. The odds ratios of seedling survival near (< 10 m) and far (> 10 m) from mature and immature large palm trees (indicated by blue circles), and female and male Myristicaceae (indicated by red squares). Each odds ratio is indicated by a point with SE error bars. Significant differences in survival of seedlings far from both classes of focal tree (far-far, indicated by a vertical dashed line) are indicated by filled points (*P *< 0.05), non-significant differences by empty points.

As expected, one-yr survival of new seedlings near (< 10 m) to mature palms was significantly lower than survival of seedlings far (> 10 m) from mature and immature palms (Figure 4). Survival of new seedlings near to immature palms was also significantly lower, but only when also near mature palms. However, unexpectedly, survival of new seedlings near to male and far from female Myristicaceae trees was significantly lower than survival of seedlings far from from sexes (Figure 4), survival of new seedlings near female trees was not significantly lower (although in these cases our analyses suffer from small sample sizes).

### Dynamic demographic effects of large trees on saplings

**Figure 5:**
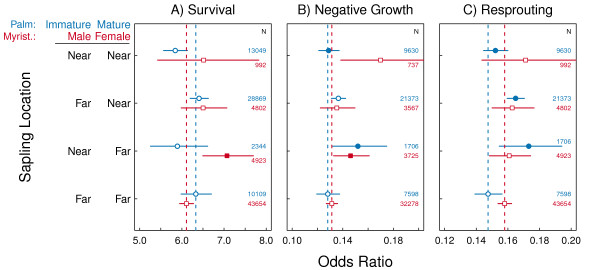
**Dynamics of saplings around large palm trees and large Myristicaceae trees**. The odds ratios of sapling survival (**A**), negative growth (**B**) and resprouting (**C**) near (< 10 m) and far (> 10 m) from mature and immature large palm trees (indicated by blue circles) and female and male Myristicaceae (indicated by red squares). Each odds ratio is indicated by a point with SE error bars. Significant differences in the response of seedlings far from both classes of focal tree (far-far) are indicated with solid points (*P *< 0.05). The vertical dashed line indicates the coefficient estimate for the baseline state (far-far).

There were some significant effects of palm and Myristicaceae trees on sapling dynamics. Contrary to our predictions, saplings showed no significant difference in the probability of survival in areas near to (< 10 m) or far from (≥ 10 m) large arborescent palms, mature or not (Figure 5A, blue circles), and the probability of negative growth of saplings near to immature palms was greater than that of saplings far from either mature or immature palms (Figure 5B, blue circles). In accordance with our predictions, resprouting rates were higher in saplings near to palm trees compared to areas far from both immature and mature palms (Figure 5C, blue circles).

When we looked at the effects of mature Myristicaceae trees on sapling dynamics, we found fewer significant effects. Sapling survival near to male but far from female Myrsticaceae trees was significantly higher compared to saplings far from both sexes (Figure 5A, red squares). However, these same saplings also had a significantly higher probability of negative growth (Figure 5B, red squares). There was no effect of Myristicaceae trees on resprouting rates (Figure 5C).

## Discussion

Using a combination of spatially-explicit static and population dynamic data, we conducted multiple tests to examine hypotheses that falling palm fronds and/or peccaries are major structuring forces of lowland Neotropical rain forests [15,36]. We found a restricted but significant direct effect of large arborescent mature fruiting palms on the spatial structure and species diversity of neighbouring sapling and seedling communities. There was also a strong and consistent larger-scale environmental effect of large palms being located in areas of lower neighbourhood density and diversity of saplings. Further, these patterns were corroborated by process in that we also found significant differences in the population dynamics of saplings (reduced growth and increased resprouting) and seedlings (lower survival) in the vicinity of mature palms. Evidence such as this was taken by previous authors to implicate falling palm fronds as major causal agents of mortality and selection in tropical forests [15]. However, when we attempted to exclude the confounding effects of frugivores by analysing still-large but immature (ie. non-fruiting) palm trees, we found no significant spatial signature, although there is some evidence of reduced growth of neighbouring saplings. This would suggest that large frugivores such as peccaries, and not falling fronds, are the more important cause of physical damage and mortality of seedlings and saplings in the vicinity of mature palms as they root around for fruit [36], because we would expect falling fronds to have an equal effect around mature and immature palm trees.

We then tested these potential effects of peccaries by examining the spatial structure and population dynamics of seedlings and saplings around another important food source, the Myristicaceae. Most species of this family are large canopy trees [54] and produce abundant crops of fruit that are eaten by peccaries [33] among other frugivores [37,38]. Unlike palms though, these trees do not have large heavy leaves that could damage seedlings and saplings beneath the canopy. Furthermore, the family is dioecious and so we could compare fruiting female trees with non-fruiting male trees. However, we found no convincing evidence that the seedling and sapling communities near female Myristicaceae were structured at all, and the significant population dynamic results we did find were in the opposite direction to that hypothesized.

Why did we find these conflicting results? One possibility is that peccaries are not large consumers of Myristicaceae fruit. It is known that peccaries consume a wide variety of species in tropical forests [33]. However, the abundance of each species in peccary diets is less well known [33]. Peccaries may focus largely on palms and eat other fruit as and when they happen upon it [32,39]. Arboreal frugivores such as toucans and primates will also likely have a large effect on the availability of fruit on the ground. Further data on peccary and other frugivore diets are required. In addition, palm seeds likely remain attractive to peccaries (if not viable) long after abscission because of their hard seed coat. Myristicaceae seeds are not protected in this way and quickly decompose.

Alternatively, there may be a difference in the pattern of abscission of palm and Myrsticaceae fruit and the seed shadows these generate. Palm fruits are held in a single infructescence at the top of the tree. These fruit then fall in rapid succession, creating a very dense carpet of fruit around the tree with a short dispersal tail [34]. Animals rooting around in this small area will likely have a big impact on the juvenile plants growing there [40]. Myristicaceae trees, on the other hand, have a much larger canopy, the fruit carpet is thinner and more spread out. Animals foraging for fruit here will likely have a lower impact on juvenile plants.

A final possibility is our assumption that the fronds of mature and immature palms are equivalent does not hold, and that immature palm trees have lighter and/or smaller leaves that cause less damage to seedlings and saplings when they fall. We were not able to compare leaves from both types of palm trees to verify this, although *Iriartea *leaves do change morphology throughout their life cycle [23,41]. Furthermore, leaves falling from shorter immature palms will have lower velocity and might do less damage than leaves falling from the greater height of mature palms. Finally, the size cut-offs determined by *Iriartea *for mature and immature individuals might be different for other species. However, these species are much less abundant than *Iriartea *and are unlikely to significantly influence our results.

### Implications for forest diversity and community structure

Given the difficulties of reliably measuring seedling growth, it was not possible to repeat all three tests of dynamic data in both seedlings and saplings. Growth and resprouting were analysed solely in saplings. However, it seems that significant mortality of seedlings occurs in the vicinity of palm trees, but that saplings can generally withstand physical damage caused by peccaries and/or palm fronds, and rather suffer reduced growth and can potentially resprout. This difference in mortality implies that different mechanisms are at work at different life history stages and that if seedlings can grow large enough, their fate is more secure [42]. The differences in spatial structure and sapling density around palms are therefore more likely to be formed at the seedling stage than the sapling stage. The small-scale repulsion of saplings (Figure 1B) may be a consequence of the immediate negative effect of large palms on seedlings (falling palm fronds cause increased mortality and stem breakage in neighboring saplings) and the large-scale effects (Figure 1A) could be a long-term cumulative population dynamics effect that results from many generations of palms having a negative impact on nearby seedlings.

The impacts of palms on the forest structure are of smaller spatial scale than that hypthesized by Peters et al. [15], who suggested that the effect of palms extended out to 7 m and that at least a quarter of lowland forest could be impacted by falling palms fronds per year. We found significant negative effects of palm trees out to only 3 m (in terms of over-dispersion of saplings [spatial pattern]) and 5 m (in terms of species repelled [ISAR]). Given the spatial pattern and density of palm trees in our plot, the area of forest predicted to be directly affected by this process is therefore reduced to 17%, although we noted above that these effects could multiply over generations leading to large areas of lower sapling density.

The negative impacts identified above appear limited to large palms and do not involve other fruiting trees such as the Myristicaceae. We would conclude therefore that this process is not a disturbance that affects large swathes of forest (or everywhere there is a tree that produces large peccary-consumed fruit). If the effect is limited to the environment of palms, this creates heterogeneity in the forest, which may lead to a wide variety of regeneration niches for species that can withstand the effects of falling leaves, fruit and rooting frugivores [43]. Once established, however, these individuals may have better access to light and soil resources, given the diffuse nature of palm canopies and the large amounts of decaying fruit and animal faeces that likely accumulate around the base of palm trees. The stilt-roots of many arborescent palms probably increase nutrient availability by trapping leaves and organic matter.

This kind of widespread small-scale disturbance creates heterogeneity in regeneration niches in both space and time. Fruiting and frugivory by peccaries is episodic, and palms generally mature one infructescence at a time. Furthermore, in the aseasonal forest of Yasuní, *Iriartea *fruit production is not confined to a few months of the year (Nancy Garwood, unpublished data). Confoundingly, palm fronds are also produced regularly throughout the year. Beck et al. [36] found no difference in leaf-fall rates between two widely separated forests in Peru. In seasonal sites such as Yasuní, therefore, where seedling germination takes place throughout the year, some individuals may escape damage simply by growing enough before the next frond or infructescence falls. In more seasonal forests where germination is often restricted to short periods, individuals are of similar ages and/or vulnerabilities and so may suffer greater impact. A further test of our hypotheses could take place in seasonal forest and examine if seedlings and saplings suffer greater mortality during the fruiting season (therefore more likely caused by peccaries) or during the non-fruiting season (more likely leaf-fall).

The ubiquitous nature of the palm family in Neotropical forests implies that given some effect, palm trees could be a powerful force structuring these forests [15]. Several species of tree dominate large areas of lowland forests in western Amazonia, and arborescent palms such as *Iriartea *are among this 'oligarch' group [24,26,28]. Widespread small-scale mosaics created by these palms may affect the composition of advanced regeneration for when a larger canopy gap does open. However, we note that peccaries are among the first large mammals to be hunted out of forest near to human habitation. Hunting will likely have dramatic effects on forest structure [32].

If reduced survival and growth around palms is predictable and selective, what can we hypothesize about species that occur in the neighbourhood of palms? Species that are better able to survive damage by falling fronds or rooting peccaries would be more likely to survive in areas close to palm trees. From our community-level analyses we cannot identify traits of individual species. However, species with underground storage organs or cotyledons might find an advantage in germinating near to a large palm because of their increased resource availability allowing resprouting following damage [15]. We identified a number of species that had significantly higher or lower abundance around palms - identification of key functional traits of these species implicated by this pattern would be useful. An advantage might even apply to the seedlings of arborescent palm trees themselves, if they can survive predation and falling fronds. *Iriartea *is one of the most widespread and abundant tree species in western Amazonia. If *Iriartea *seedlings can survive better in the adult zone of influence than the majority of non-palm species, this may go some way to explaining how such abundant populations can be maintained. Future work should therefore look for more evidence of selection potential of the process that Peters et al. [15], Beck et al. [36] and we have identified. What species are selected for? Does species diversity change predictably from seedlings to saplings? Are there species that appear more frequently together in proximity to palms than in other parts of the forest? Furthermore, a broader-scale comparison with other forests may be helpful. For example, the forests of SE Asia are not dominated by palms as are Neotropical ones, and yet peccaries have a significant impact on seedling and sapling communities [31].

Palms themselves are not immune to the effects of branch falls [44] and suffer negative density dependence [45], caused by falling fronds [22,46-48], peccaries [32,34], and pathogenic fungi [49]. Although palm trees significantly impact non-palms, the combination of falling fronds and peccary seed predation and fungal attack inhibit population growth such that palms may currently be at maximum density in the forest.

## Conclusions

On a much larger scale then previously tested, we examined hypotheses that peccaries and/or falling palm fronds are major structuring forces of lowland Neotropical rain forests. We found a restricted but significant direct effect of large arborescent mature fruiting palms on the spatial structure, population dynamics and species diversity of neighbouring sapling and seedling communities. These effects were not found in smaller non-fruiting palm trees. However, this evidence of a primary effect of peccaries on the seedling and sapling communities around palms was not supported in data from other edible species in the Myristicaceae.

Given the abundance of arborescent palm trees in Amazonian forests, it is reasonable to conclude that their presence does have a significant, if spatially-restricted, impact on juvenile plants. The widespread effects of these small-scale disturbances appear, over long time-scales, to cause directional changes in community structure. However, the population growth of large arborescent palms themselves is very likely limited by the very same two factors that impact their surrounding juvenile plant communities.

## Methods

### Tree and sapling data

A 25-ha permanent forest dynamics plot (FDP; http://www.ctfs.si.edu) is located inside Yasuní National Park (0°41S, 76°24W; [50]), a still largely wilderness, tropical lowland aseasonal rain forest in eastern Ecuador [51]. Mean annual rainfall is approximately 2800 mm and total monthly rainfall is almost never ≤ 100 mm. Mean monthly temperature is 25-27°C [50]. The FDP ranges from 216 to 248 m a.s.l: it includes two ridges and an intervening valley that floods for brief periods. From 1995-1999, all freestanding stems ≥ 1 cm diameter at breast height (dbh at 1.3 m), excluding lianas, were tagged, mapped and identified [50]. In the first census, 1104 morph species were recorded, comprising 151,230 individual trees [50]. The FDP was recensused in 2002-2003 [52].

There are four species of arborescent palm in the Yasuní FDP: *Astrocaryum chambira *Burret, *Iriartea deltoidea *Ruiz & Pav., *Oenocarpus bataua *Mart., and *Socratea exorrhiza *(Mart.) H. Wendl. The majority of individuals are *Iriartea *(Table 1). Although the relationship between stem diameter and height in palms is not as robust as that in dicotyledon trees, *Iriartea *generally reaches maturity around 15 cm dbh [53]. Because the height of palms is not measured on the FDP, we took this diameter as the cut-off between mature and immature large palms. In order to examine the interaction between frugivory and leaf-falls, we compared mature fruiting palms (> 15 cm dbh) to large palms without fruit, being those 10-15 cm dbh.

The size at which tropical trees mature is difficult to examine and often unknown for many species and can depend on a number of factors such as light availability, nutrient and water resources. However, the Myristicaceae at Yasuní have been studied intensively for the previous 10 years, and trees have been visited multiple times to look for signs of reproduction. The family is dioecious and all reproductive (ie. mature) male and female trees have been identified and sexed from flowers and/or fruit [54-58]). The environment below a male and female Myristicaceae adult should be qualitatively similar, allowing the separation of the effect of fruit crop and associated frugivores from the effects of light availability or habitat quality on the performance of seedlings and saplings.

### Seedling data

Within the 25-ha FDP, 339 1 m^2 ^seedling plots were established along trails in 2002 [59]. They were arranged around seed traps in sets of three as part of a long-term community phenology study. Survival, growth and recruitment are monitored every year and we use data from 2003 to 2008.

#### Data analysis

We conducted two sets of analyses. First we looked at the static spatial patterns of saplings and trees in the FDP. Second we examined the demographic rates of seedlings and saplings to understand the causes of these patterns. In all analyses, we tested the previously identified potential drivers of spatial patterns and demography. These were (i) large mature arborescent palms > 15 cm dbh, (ii) smaller, immature arborescent palms 10-15 cm dbh, (iii) large mature female Myristicaceae trees, (iv) large mature male Myristicaceae trees. In comparisons examining palms, differential responses based on proximity to larger or smaller palms are likely due to the effects of fruiting, while similar responses could be attributed to large, falling fronds common to both groups. Comparisons between male and female Myristicaceae isolate the effects of a large fruit crop and may generalize impacts of peccaries, if any exist, of the large palms. Unless otherwise stated, all analyses were conducted with R [60].

### Spatial patterns of distribution and density

In order to examine patterns of attraction and repulsion between two sets of spatially-explicit points, we used an extension of the O-ring statistic for bivariate patterns [61] to test, for example, the density of saplings (1-2 cm dbh) at various distances *r *around large palms (> 15 cm dbh). More formally, the O-ring statistic *O_12_(r) *for a bivariate pattern composed of type 1 and type 2 points yields the average density of type 2 point within rings of radius *r *and width *dr *around the points of pattern 1. The *O_12_(r) *is also called neighbourhood density function and is closely related to the pair correlation function *g_12_(r)*, i.e., *O_12_(r) = λ*2*g*_12_*(r) *[61,62] where λ2 is the average density of type 2 points in the plot.

To find out if the distribution of saplings in the neighbourhood of palms or Myristicaceae trees is different from a distribution expected by chance for each of these analyses we initially tested two different null models of spatial distribution. The first null model retained the spatial distribution of saplings and assumed a random distribution of palms (i.e., a homogenous Poisson null model [61]). This null model therefore compares the observed neighbourhood density of saplings around palms with sapling densities in random locations. If the neighbourhood density of saplings around focal trees is smaller (or larger) than expected for randomly relocated focal trees we can infer a negative (or positive) impact of focal trees on saplings. However, we could observe the same pattern if (larger-scale) environmental heterogeneity acted such that the focal trees were located in areas of higher or lower overall sapling density. We therefore used a second null model that approximately factors out such larger-scale environmental variability and reveals the "pure" smaller-scale effects of focal trees on saplings (e.g. [63-65]). The second test also assumed a random distribution of palms, but the palms were only displaced within a given neighborhood of 30 m. The neighborhood of 30 m is somewhat larger than the expected range of direct interactions between focal trees (i.e., palms and adult Myristicaceae trees) and saplings (< 10 m based on ePeters2004). Technically, this null model was implemented as a heterogeneous Poisson process with a non-parametric kernel estimate of the intensity function λ1(*x*) of the focal trees (for details see [64]).

We made these comparisons for (i): non-palm sapling distributions versus mature and immature palms and (ii) non-palm sapling distributions versus male and female Myristicaceae. All analyses were run for 199 simulations and 95% simulation envelopes were calculated. These analyses were conducted using the Programita software package [61].

### Spatial patterns of community structure

While the previous analysis focused on overall sapling density we also carried out tests where we compared the species diversity of sapling communities that were influenced by palms to sapling communities that were not. For the first two tests we compared sapling (1-2 cm dbh) communities in 5 × 5 m quadrats that either contained ≥ 1 palm or were adjacent to a quadrat containing a palm to quadrats that were > 1 quadrat distant from a palm-quadrat. This accounted for the up to 10 m distance over which palm fronds fall. In the third test, we examined the individual species-area relationships between palms and saplings.

First, we tested whether sapling density, species richness and rarefied species richness per two individuals were lower in quadrats influenced by palms, using the 'vegan' R package [66]. This provided a basic description of the community spatial structure with minimal consideration of species identity.

Second, we compared the community composition of saplings in quadrats using a multiple response permutation procedure (adonis), in 'vegan' [66]. This method is analogous to a multivariate analysis of variance and provides a robust test of whether there is a significant difference between the species composition of palm and non-palm quadrats. Palm and non-palm quadrats may have equivalent species richness yet these species need not be identical. We also compared the abundance of every species in the plot in palm and non-palm quadrats using a proportion test, with the expected relative abundance set to 85% of individuals in non-palm quadrats (the proportion of all quadrats that were non-palm). Species with significantly higher or lower abundances than expected (*P *< 0.05) were identified.

Third, we examined the individual species area relationships (ISAR; [64]) for neighbourhoods around individual large palms. The ISAR unites spatial patterns and species identity. It allows for a much subtler assessment of species effects on local diversity with respect to their interactions with plants of other species than the two previous methods because it considers each interspecific interaction rather than the focal species versus all species pooled. The *ISAR(r) *function is the expected number of species within radius *r *around an arbitrary individual of a target species. Technically, the ISAR function can be expressed for a given focal species *f *as sum of nearest neighbor distribution functions *D_fi_(r) *because the probability that a given individual of species *i *is within distance *r *of an individual of the focal species *f *is given as 1-*D_fi_(r)*:

(1) $$ISAR_{f}\left(r\right)=\overset{S}{\underset{i=1}{\sum }}\left(1-D_{\mathrm{fi}}\left(r\right)\right)$$

*ISAR *is a statistic to analyse the spatial diversity structure in forest ecosystems and reconciles common species area relationships [67-69] and the individual perspective of point-pattern analysis [63,70]. The *ISAR *allows for a subtle assessment of the effect of palms (or adult Myristicaceae) trees on the non-palm sapling diversity in their neighbourhood. If negative interactions of the focal species on saplings dominate, the target species would decrease the proportion of species in its neighborhood (i.e., a diversity repeller). Conversely, if the target species exerts positive interactions to non-palm saplings, the target species would accumulate and maintain an over-representative proportion of sapling diversity in its proximity (i.e., being a diversity accumulator). Finally, if positive and negative interactions are weak or even out, the focal species would behave neutrally.

Similar to the case of the neighbourhood density described above, environmental heterogeneity may cause repeller or accumulator effects in the absence of species interactions if the focal species is predominantly located in areas of low or high sapling diversity. We therefore contrasted the observed *ISAR(r) *function to that of the same null models as for the analyses of neighbourhood density. In the first null model we distributed individuals of palms randomly over the entire plot. The second test also assumed a random distribution of palms, but the palms were only displaced within a given neighborhood of 30 m to remove in the null model potential interactions with saplings and to guarantee that the displaced palms will be as well located in areas with low sapling density. The neighborhood of 30 m is somewhat larger than the expected range of direct interactions between focal trees. These analyses were conducted in Programita, for more details of *ISAR*, see Wiegand et al. [64].

We hypothesize that large palms are a diversity repeller because of the disturbance effects of falling fronds or rooting damage by palm fruit consumers in the vicinity of palms. Only a subset of species would have the traits necessary for resprouting and continued growth following damage from nearby palms.

### Dynamic demographic effects of large trees on seedlings

To examine the effects of proximity to large palms and Myristicaceae on seedling survival, for each of the 339 seedling plots located in the FDP we calculated the total number of new recruits of all species between 2003 and 2007. We then calculated the number of each cohort surviving one year. We modelled the proportion of survivors in each plot as a function of location relative to mature and immature large trees with a generalised linear model with binomial errors as above.

### Dynamic demographic effects of large trees on saplings

We examined the survival, growth and resprouting of saplings 1-2 cm dbh from the first to the second plot census as a function of whether the sapling was near to (within 10 m) or far from (> 10 m) focal trees (palm or Myristicaceae). First, we modelled the probability of survival to the second census of all saplings alive in the first census. Second, we modelled the probability of each sapling resprouting (new sprouts growing up and out from damaged or broken stems). Third, growth rate for each sapling from the first census to the second census was calculated as growth = (dbh1 dbh0)/(time1 time0) and divided into negative (1) or ≥ zero growth (0). We modelled the probability of a negative growth rate between censuses. In all three cases we used a generalised linear model with binomial errors. We modelled survival (or growth or resprouting, all binary response variables) as a function of proximity to the nearest focal tree (mature and immature palm or male and female Myristicaceae): survival ~ mature palms * immature palms. We coded each sapling as near or far (</> 10 m) from focal trees. We included the interaction term because saplings could be near to a mature palm but far from an immature palm, for example.

## Authors' contributions

SAQ conceived the study, carried out the statistical analyses and drafted the manuscript. MRM participated in the design of the study and helped draft the manuscript. TW oversaw the spatial analyses and interpretation. RV helped conceive and design the study. All authors contributed to, read, and approved the final manuscript.
